# Exploring Orientation Invariant Heuristic Features with Variant Window Length of 1D-CNN-LSTM in Human Activity Recognition

**DOI:** 10.3390/bios12070549

**Published:** 2022-07-21

**Authors:** Arnab Barua, Daniel Fuller, Sumayyah Musa, Xianta Jiang

**Affiliations:** 1 Department of Computer Science, Faculty of Science, Memorial University of Newfoundland, St. John’s, NL A1C 5S7, Canada; abarua@mun.ca; 2 School of Human Kinetics and Recreation, Memorial University of Newfoundland, St. John’s, NL A1C 5S7, Canada; dfuller@mun.ca (D.F.); sbmusa@mun.ca (S.M.)

**Keywords:** human activity, CNN, LSTM, window length, inter-participant evaluation, orientation invariant, accelerometer, smartphones

## Abstract

Many studies have explored divergent deep neural networks in human activity recognition (HAR) using a single accelerometer sensor. Multiple types of deep neural networks, such as convolutional neural networks (CNN), long short-term memory (LSTM), or their hybridization (CNN-LSTM), have been implemented. However, the sensor orientation problem poses challenges in HAR, and the length of windows as inputs for the deep neural networks has mostly been adopted arbitrarily. This paper explores the effect of window lengths with orientation invariant heuristic features on the performance of 1D-CNN-LSTM in recognizing six human activities; sitting, lying, walking and running at three different speeds using data from an accelerometer sensor encapsulated into a smartphone. Forty-two participants performed the six mentioned activities by keeping smartphones in their pants pockets with arbitrary orientation. We conducted an inter-participant evaluation using 1D-CNN-LSTM architecture. We found that the average accuracy of the classifier was saturated to 80 ± 8.07% for window lengths greater than 65 using only four selected simple orientation invariant heuristic features. In addition, precision, recall and *F*1-measure in recognizing stationary activities such as sitting and lying decreased with increment of window length, whereas we encountered an increment in recognizing the non-stationary activities.

## 1. Introduction

Human activity recognition (HAR) has allowed for the implementation of distinct applications such as user identification [1], health monitoring [2], identifying the early stage of depression [3], fall detection [4], and more. Improving these applications requires ongoing methodological development. Researchers have conducted many studies to improve HAR by introducing the recognition of various daily activities using divergent approaches that include non-identical machine learning algorithms. Improving HAR requires considering some inevitable challenges, which involve sensor orientation, sensor position, device independency, study sample length, and data volume [5,6,7,8]. Among the mentioned challenges, the most significant issue to solve is the problem of sensor orientation and position.

For solving the orientational and positional problem due to sensor placement, different studies have introduced techniques such as transforming the sensor signals to a universal frame, extracting orientation-invariant features from raw signals, removing orientation and position-specific information by introducing statistical alterations, estimating the orientation of the sensor to the earth frame by using the triaxial sensors (accelerometer, magnetometer, and gyroscope), and then transforming the raw signals from the sensor frame to the earth frame. Using the earth frame transformation approach [9] achieved an average accuracy of 86.4% in recognizing 19 activities using support vector machine (SVM). The authors of [10] introduced heuristic orientation invariant transformation and singular value decomposition-based transformation to tackle sensor orientation problems. They evaluated their approaches using 4 different classifiers on 5 distinct datasets. They found that their proposed approaches can reduce the accuracy drop by a considerable margin compared to other state-of-the-art approaches. The authors of [11] decomposed the accelerometer signal into horizontal and vertical acceleration. They extracted nine features from the triaxial gyroscope sensor and horizontal and vertical acceleration signals to solve the position and orientation dependency problem. They acquired an accuracy of 91.27%, employing SVM to recognize 5 activities using the data from a smartphone in 4 different positions. For our study, we decided to utilize the features proposed by [10] since it requires extracting nine simple features to eliminate the variation in data produced from sensor orientation and position. Along with the orientational and positional dependency obstacles, we should also consider the number of participants and activities appraised in former studies.

As the number of participants and activities varies, distinct variations in sensor signals appear due to the differences in the participants’ body attributes and the uniqueness in movements of the body parts during different activities. The number of participants matters, especially for the studies where the inter-participant evaluation technique is accepted as the validation method. There are many publicly available datasets to work with, and these have already been used in several studies that introduced different numbers of participants and activities [12,13,14,15,16]. However, inter-participant evaluation for a large number of participants in the field of HAR is yet to be explored. 

Regarding the employed classifiers for HAR, researchers evaluated the performance of both machine learning and deep learning algorithms. Research primarily assessed the performance of conventional machine learning algorithms such as support vector machine (SVM), decision tree, K-nearest neighbor, and random forest [17,18,19,20]. However, with the emergence of advanced computational power, deep learning algorithms became more common in HAR. The authors of [12] evaluated the performance of the convolutional neural network (CNN), long short-term memory (LSTM), bidirectional-LSTM, and multilayer perceptron (MLP) using two public datasets named UCI [21] and Pamap2 [22]. They found that CNN outperformed other classifiers with 92.71% and 91% accuracy on UCI and Pamap2, respectively. The authors of [23] compared CNN with state-of-the-art classifiers for classifying six activities and showed that CNN performed better than all other classifiers using features extracted by fast Fourier transform (FFT) with an accuracy of 95.75%. CNN remains favored for executing HAR because of its powerful ability to automatically extract features from raw signals using multiple filters [24]. Studies then tried to combine the feature extraction power of CNN with LSTM’s power of persisting old information about time-series data. LSTM is an upgraded version of the recurrent neural network (RNN) that can preserve older information than RNN [25]. The hybrid of CNN and LSTM, also called CNN-LSTM, has been used in different HAR studies. The authors of [26] evaluated the performance of CNN-LSTM on HAR using three public datasets named UCI [21], WISDM [27], and OPPORTUNITY [28]. They achieved accuracies of 95.78%, 95.85%, and 92.63% on UCI, WISDM, and OPPORTUNITY datasets, respectively, using a CNN-LSTM architecture. The authors of [29] explored distinct deep learning architectures, including CNN-LSTM with their proposed margin-based loss function on OPPORTUNITY, UniMiB-SHAR [15], and PAMAP2 datasets. The authors of [30] ensembled three models, namely CNN-Net, Encoded-Net, CNN-LSTM, and found the performance of the ensembled model superior over six benchmark datasets. 

There are a number of different implementations of CNN for sensor data. Typically, 1-dimensional CNN (1D-CNN) is used for accelerometer, gyroscope, and magnetometer signals. An important consideration with 1D-CNN, LSTM, or their hybrid is that these methods require data windows as inputs. Each window resembles a data matrix with a fixed number of samples as rows and the features as columns. Each consecutive window may or may not overlap. 1D-CNN uses filters on each window to extract features automatically. 1D-CNN maps these internally extracted features to different activities in HAR research. However, when 1D-CNN is combined with LSTM, the internally extracted features from 1D-CNN work as inputs to the LSTM layers. These LSTM layers further process these automatically extracted features. The advantage of using a 1D-CNN-LSTM hybrid rather than using a single CNN or single LSTM is that 1D-CNN-LSTM can use the ability of CNN to extract spatial features present in the input data as well as preserve the temporal information present in the extracted spatial features using the ability of LSTM. Although a 1D-CNN-LSTM system takes more time in training than a single CNN, it should not impose any problem in the deployment of real-life applications since, in real life, pre-trained models are deployed. A detailed explanation of the working mechanism of 1D-CNN-LSTM will be given in a later section. Now that 1D-CNN works with windows of data, the length of windows can affect the performance of 1D-CNN. With a large window length, the model will have a bigger picture of the signals’ nature at a particular time. In contrast, a smaller window length portrays comparatively less information regarding the signal nature at any specific time. Again bigger windows increase the computational complexity and time complexity, whereas smaller windows keep the computational burden and processing time considerably lower. Previous studies selected the window length arbitrarily in HAR execution while using CNN, LSTM, or their hybridization, or they did not provide any discussion regarding the selection of window length [23,31,32,33,34,35]. It is yet to be explored how different window lengths may affect the performance of CNN, LSTM, or their hybrid models in HAR research.

**Figure 1:**
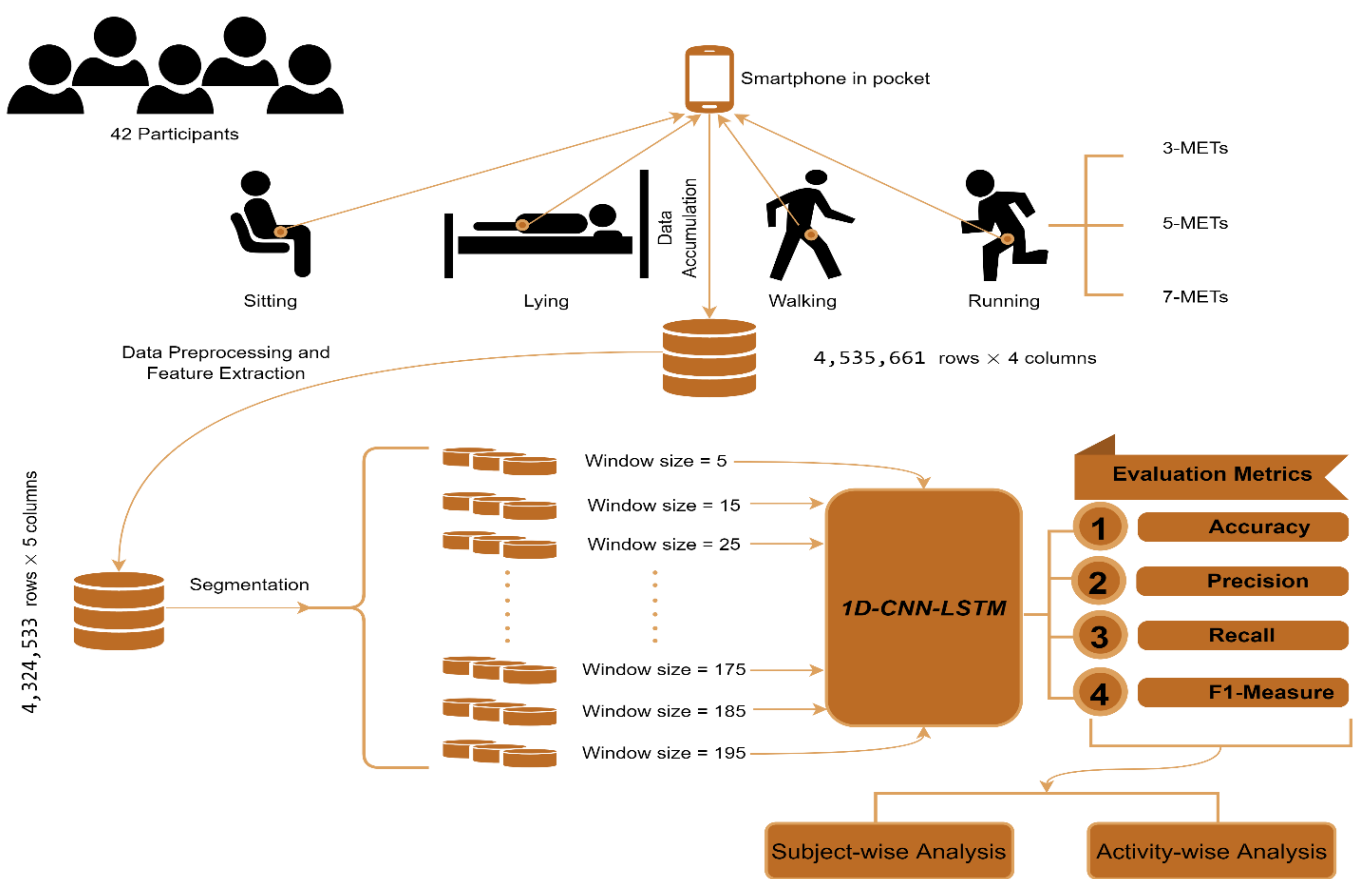
Structural diagram of our overall study.

Considering the shortcomings mentioned above, the orientation problem, study sample lengths, and window length considerations, this paper systematically examines these limitations using feature extraction methods and window length experiments with 1D-CNN-LSTM models. A pictorial view of the overall procedure is portrayed in Figure 1. Data from a study including 42 participants performing 6 activities, namely, sitting, lying, walking, and running at 3 metabolic equivalent tasks (METs), 5 METs, and 7 METs pace, were used. Data were collected using an accelerometer sensor of a smartphone carried by participants in their pockets. Specific research questions were:

- How can sensor orientation be solved?

To solve the sensor orientation problem due to the flippable positions of the smartphone in the pocket, we selected 4 orientation-invariant heuristic features from the proposed 9 heuristic features in [10].

- What is the impact of window length on model accuracy?

Results show that after a particular window length, the performance of 1D-CNN-LSTM is not influenced by the window length. Further examination explores how different window lengths influence the recognition metrics for high- and low-intensity activities.

- What is the impact of the inter-participant validation method in the case of a vast number of participants?

We found that the model did not produce the same performance when evaluated using data from different participants. Still, the effects of window length on the performance of the different participants were the same.

## 2. Materials and Methods

In this section, we will start by discussing the data accumulation process. Then we will explain the data preprocessing step and features we extracted to solve the smartphone orientation problem. In the following section, we will present the structure of the 1D-CNN-LSTM we employed in our study to discuss its effectiveness briefly. We present the discussion regarding the window lengths and their impact in the next section.

### 2.1. Data Accumulation 

We acquired verbal and written consent from 42 healthy participants aged 18 years and older to collect the required data. Before participating in the protocol, the participants completed the physical activity readiness questionnaire (PAR-Q). We acquired the necessary ethical approvals from the Memorial University Interdisciplinary Committee on Ethics in Human Research (ICEHR #20180188-EX). A summary of the demographics (gender, age, height, and weight) is presented in Table 1. We did not include the participants’ demographics as attributes in our dataset because, in another study [36], we found that these attributes did not significantly influence the performance of the machine learning models.

Each participant carried a Samsung Galaxy S7 (SM-G930W8) smartphone in their pocket with a pre-installed Ethica application [37] that recorded the *X*, *Y*, and *Z*-axis values of the accelerometer sensor embedded in the mentioned smartphone while completing the protocol. The lab-based protocol required 65 minutes for each participant to complete entirely. During the 65 minutes, each participant conducted the activities according to the order and for the time presented in Table 2. The rank column in Table 2 shows the order of the activity trial. Rank 1 means each participant started with the corresponding activity trial, and rank 9 points to the activity trial that each participant completed at the end. Participants had the freedom to keep the smartphone in their pocket in any orientation.

The participants walked and ran on a treadmill set up in the lab. To measure the intensities of running, we used the metabolic equivalent of task (MET) [38], a relative measure of energy expenditure related to the participant’s weight and volume of oxygen consumed per minute. We used MET rather than walking speed, cadence, or stride length because we wanted to quantify the intensity of activities using energy expenditure. For the same walking speed, cadence, or stride length, we may record different energy expenditures from different participants. Furthermore, MET has been highly recommended by other studies to measure energy expenditure. Equation (1) defines the calculation process of MET.

$$\mathrm{MET}=\frac{\mathrm{Oxygen}\;\mathrm{Cosumption}\;\mathrm{rate}\;\left(\frac{\mathrm{mL}}{\mathrm{minute}}\right)}{3.5\times \mathrm{weight}\;\left(\mathrm{kg}\right)}$$

Here mL is a unit of volume of oxygen that stands for milliliter, and kg stands for kilogram, which is a unit for measuring the weight. 

The reason for choosing the aforementioned activity types and activity intensities was that these were reported to be the most common type of activities included in former studies [39]. Besides, walking or running with different intensities were overlooked in most of the former studies. Therefore we focused on studying the effects of window length for 1D-CNN-LSTM on HAR for common types of activities and intensities.

### 2.2. Data Preprocessing and Feature Extraction

We used programming languages R 3.6.1 and Python 3.9.7 to execute the required preprocessing of the data and extract heuristic features, respectively. We used Python packages named Pandas 1.3.4 and Numpy 1.20.3 for performing feature extraction. 

#### 2.2.1. Data Resampling and Data Imputation

The Ethica App could not accumulate the sensor data in a constant frequency; rather, the frequency varied from 5 Hz to 19 Hz. The reason behind this varying frequency was the application’s forced optimization technique to keep the app running by constraining the amount of data uploaded to the server. Because of this varying frequency, each activity class had a very different number of observations, although they should have an almost similar amount of observations. We upsampled the data to a constant frequency of 30 Hz by using the resampling method introduced in [33] to eliminate this data imbalance. In addition, we also experienced missing values in our accumulated data. This problem happened due to the momentary connection loss between the Ethica App and the server. To get rid of the problem regarding these missing values, we conducted data imputation. For performing linear imputation, we used the R package named ImputeTS. We then performed the process of feature extraction. After data resampling, imputation execution, and feature extraction, we had the following amount of data points for each activity presented in Table 3. 

#### 2.2.2. Feature Extraction and Selection

**Figure 2:**
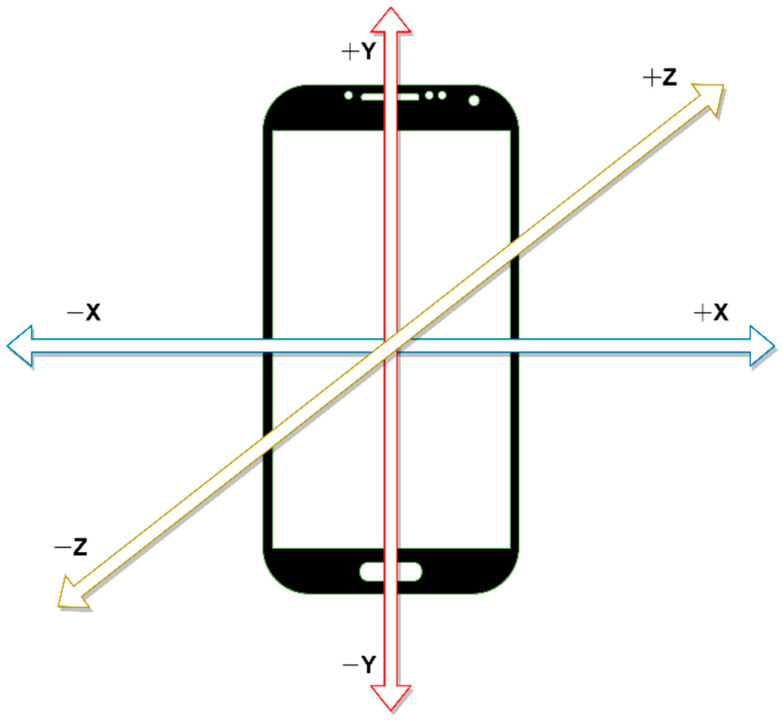
The direction of three axes of an accelerometer sensor in a smartphone.

We extracted some suitable heuristic features to resolve the sensor orientation problem. As mentioned earlier, during the data collection phase, the participants had the freedom to keep the mobile phone in their pocket in any arbitrary orientation. Therefore, different participants might perform the same activity trial while keeping the smartphone in a non-identical orientation. If we observe Figure 2, we can see the direction of the axes of the accelerometer with respect to the smartphone. The *X*-axis goes from left to right of the smartphone screen, *Y*-axis goes from top to bottom, and *Z*-axis goes perpendicularly through the screen. As we orient the smartphone, the axes are also oriented accordingly. As a result, we observed different accelerometer *X*, *Y*, and *Z*-axis values for the same activity if the users kept the smartphone in different orientations while performing the same activity. 

**Figure 3:**
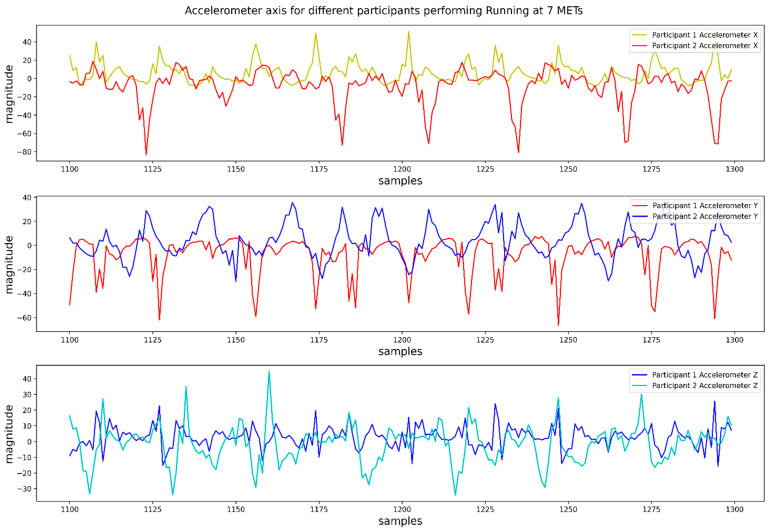
Values of three accelerometer axes for the same activity performed by two different participants.

If we observe Figure 3, we can see how the values of the axes of the accelerometer differ in values while different participants ran at a speed of 7 METs. To solve this problem, we extracted 4 simple heuristic features which were proposed in [10]. This study originally proposed 9 sensor invariant heuristic features. It defined each data vector of the accelerometer as $\vec{v_{n}}=\left(v_{x}\left[n\right],\;v_{y}\left[n\right],\;v_{z}\left[n\right]\right)$ where $v_{x}\left[n\right]$, $v_{y}\left[n\right]$, $v_{z}\left[n\right]$, were values of accelerometer *x*-axis, *y*-axis, and *z*-axis, respectively, at any time sample n. They also defined first-order and second-order time differences as $\Delta \vec{v_{n}}=v_{n+1}-v_{n}$ and $\Delta^{2}\vec{v_{n}}=v_{n+1}-v_{n},$ respectively. The equations for computing the 9 heuristic features are given below,

(1) $$w_{1}\left[n\right]=\left\|\vec{v_{n}}\right\|$$

(2) $$w_{2}\left[n\right]=\left\|\Delta \vec{v_{n}}\right\|$$

(3) $$w_{3}\left[n\right]=\left\|\Delta^{2}\vec{v_{n}}\right\|$$

(4) $$w_{4}\left[n\right]=∠\left(\vec{v_{n}},\;\vec{v_{n+1}}\right)$$

(5) $$w_{4}\left[n\right]=∠\left(\Delta \vec{v_{n}},\;\Delta \vec{v_{n+1}}\right)$$

(6) $$w_{4}\left[n\right]=∠\left(\Delta^{2}\vec{v_{n}},\;\Delta^{2}\vec{v_{n+1}}\right)$$

(7) $$w_{7}\left[n\right]=∠\left(\vec{p_{n}},\;\vec{p_{n+1}}\right)\;\mathrm{where}\;\vec{p_{n}}=\vec{v_{n}}\times \vec{v_{n+1}}$$

(8) $$w_{8}\left[n\right]=∠\left(\vec{q_{n}},\;\vec{q_{n+1}}\right)\;\mathrm{where}\;\vec{q_{n}}=\Delta \vec{v_{n}}\times \Delta \vec{v_{n+1}}$$

(9) $$w_{9}\left[n\right]=∠\left(\vec{r_{n}},\;\vec{r_{n+1}}\right)\;\mathrm{where}\;\vec{r_{n}}=\Delta^{2}\vec{v_{n}}\times \Delta^{2}\vec{v_{n+1}}$$

Here,

$$w_{t}=extracted\;heuristic\;features\;for\;t=1\;to\;9$$

$$\|\vec{m}\|=Euclidean\;norm\;of\;vector\;m$$

$$∠\left(\vec{a},\;\vec{b}\right)=cos^{-1}\left(\frac{\vec{a}\;\cdot \;\vec{b}}{\;\|\vec{a}\;\vec{b}\|}\right)=angle\;between\;vector\;a\;and\;vector\;b\;where\;\vec{a}\;\cdot \;\vec{b}\;denotes\;their\;dot\;product$$

**Figure 4:**
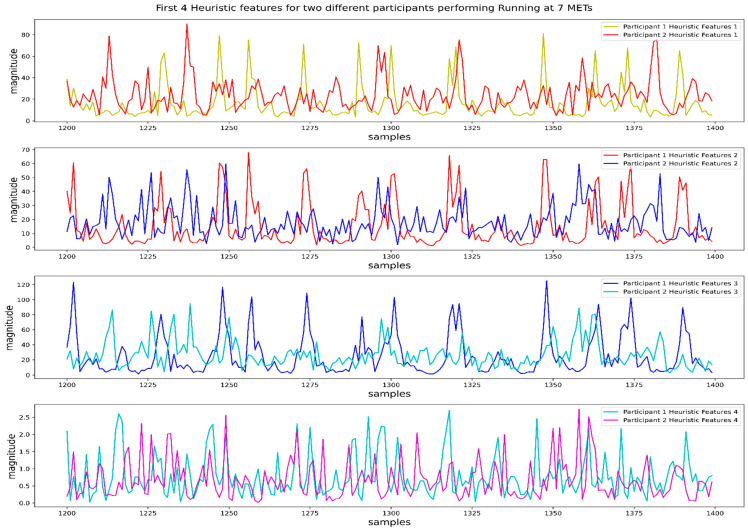
Values of the first four heuristic features for activity running at 7 METs from two different participants.

They claimed these 9 heuristic features to be irresponsive to the orientation of the sensor and mathematically elaborated on the reason behind being invariant to the orientation. Further analysis can be found in [10]. Although this study examined the performance of all 9 features in HAR execution, we conducted a more detailed analysis of these features. We found that the first 4 features, $w_{1},\;w_{2},\;w_{3,}$ and $w_{4,}$ are most important and distinguishable for different activities. In Figure 4, we can observe the pattern and magnitude range of the first 4 heuristic features for two participants performing the same running activity at a speed of 7 METs. We can observe some similarities in the pattern and magnitude range of 4 heuristic features. 

**Figure 5:**
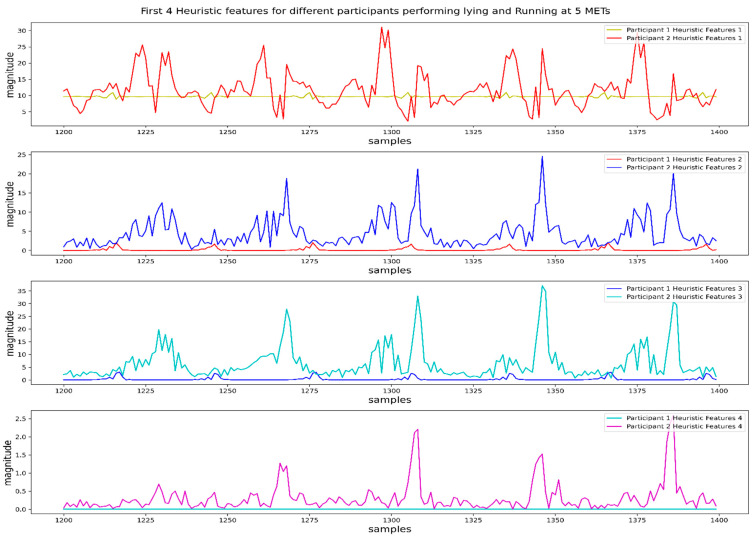
Values of the first four heuristic features for two different activities from two different participants. Participant 1 performed activity lying, and participant 2 performed activity running at 5 METs.

In Figure 5, we plot the values of the first 4 heuristic features for different participants performing two different activities named lying and running at a speed of 5 METs. We can observe a considerable difference in the patterns and magnitude of the first 4 features for two different activities performed by two different participants. That means the first 4 heuristic features showed similarities in their values for the same activity and dissimilarities for different activities, which is essential for any classifier to distinguish them.

In addition, we computed the feature importance of all 9 features using the two classifiers named decision tree (DT) and random forest (RF). Here, feature importance defines how impactful a feature is to a classifier to make a prediction. We chose these two classifiers for computing feature importance because they are proven to be very effective. There are different versions of DT. We used the version named classification and regression tree (CART). DT and RF have two metrics to decide the importance of features, named Gini impurity and information gain. 

**Figure 6:**
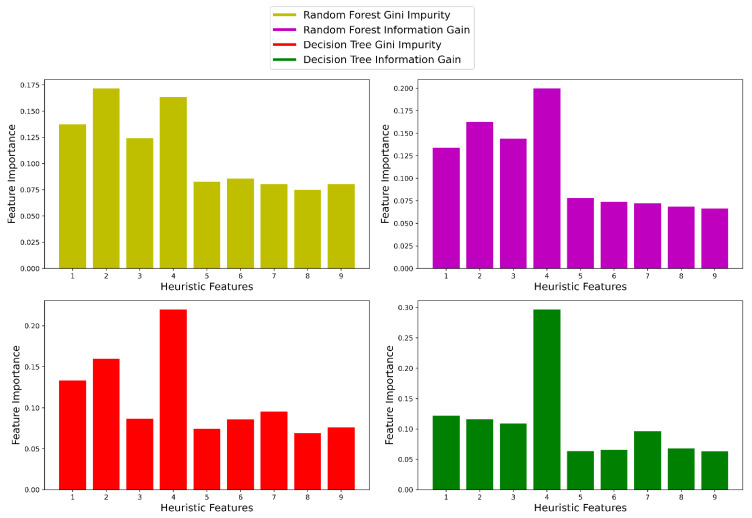
Feature importance of the nine heuristic features.

Our study used both as metrics to calculate the feature importance. Although RF uses multiple decision trees to compute its result and is comparatively better than DT, there are still differences between DT and RF regarding how they use the whole dataset. Therefore, we wanted to compute feature importance using both classifiers because of their different nature. For the functioning of these classifiers in Python 3.8.10, we used their implementation provided in the package named scikit-learn 0.22.1. We fed the whole dataset to both classifiers to calculate the feature importance. Feature importance for the 9 features is depicted graphically in Figure 6. 

From Figure 6, we can see that the first four features had greater feature importance than the last 5 heuristic features when we used RF using Gini impurity and information gain to compute the feature importance. In the case of DT, although it assigned greater feature importance to the first 4 heuristic features using Gini impurity and information gain; it also assigned considerable importance to the 7th feature. However, considering all four scenarios of feature importance, we elected to use the first four features as they were assigned greater feature importance in all cases. We also wanted to find if scaling the data had an impact on feature selection, which is why we computed the feature importance after scaling the features between 0 and 1. The result did not change. Even after scaling, the first 4 heuristic features had superior feature importance. Since we used the raw heuristic features for our study, we did not present the feature importance found after scaling the data in this paper.

### 2.3. The Architecture of 1D-CNN-LSTM

**Figure 7:**
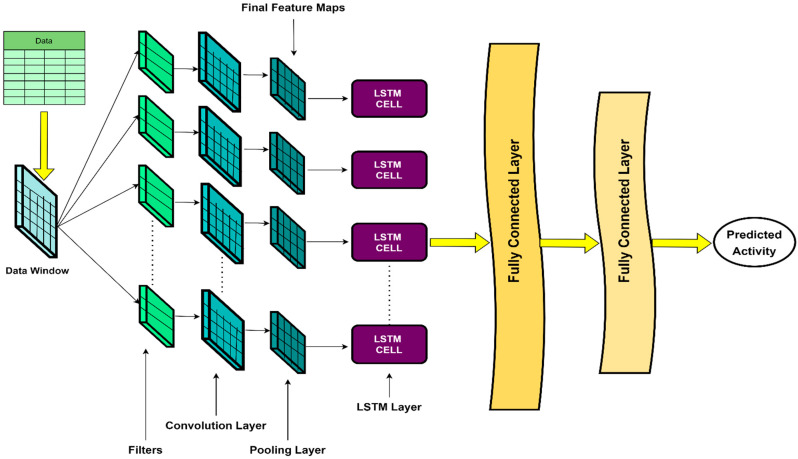
Proposed structure of the 1D-CNN-LSTM model.

A conventional CNN consists of an input, convolution, pooling, fully connected, and output layer. The input layer takes the data matrix as input. A data matrix encapsulates a portion of the data. The convolution layer consists of multiple filters, where each filter is also a matrix with lower dimensions than the fed data matrix. Each filter can move on the input data matrix in two directions or one direction for 2D-CNN and 1D-CNN, respectively. Each filter performs a convolution operation and constructs a feature map when moving on the input data matrix. A convolution layer with *n* number of filters constructs *n* number of feature maps for a single data matrix. We can define a feature map as a representation of the original data matrix with equal or lower dimensions but concentrate on prioritizing a particular feature of the data matrix. The pooling layer reduces the length of feature maps by moving the averaging filter or max filter on them. We can feed these feature maps into an LSTM model. An LSTM model [25] can have one or more LSTM layers. Each LSTM layer consists of multiple LSTM cells. Each cell encapsulates three gates named forget gate, input gate, and output gate. The forget gate is responsible for removing unnecessary information from the previous time step where the time step resembles a row in a data matrix. The input gate process the current data fed into the cell, and the output gate generates the output to be combined with the next input to the LSTM cell. In our hybrid 1D-CNN-LSTM [40], we had convolution and pooling layers followed by an LSTM and fully connected layers, as depicted in Figure 7. The final pooling layer generates final feature maps fed into LSTM cells. The LSTM cells then consider each feature map as a time step and try to learn from it and propagate the information to use it in processing the next feature map (time step). The LSTM layer’s output then goes to the fully connected layer, composed of conventional neurons of an artificial neural network (ANN). The predictive result comes out of the final fully connected layer.

Our proposed 1D-CNN-LSTM architecture started with a CNN encompassing 6 convolutional layers, 3 pooling layers, and 4 dropout layers. The convolution layers used different kernel lengths and rectified linear unit (relu) as their activation function. We introduced dropout layers in the model to reduce the risk of overfitting. For LSTM‘s portion, we used an LSTM layer with 512 hidden units and tanh as its activation function. Four fully connected dense layers followed the LSTM layer. The first 3 layers had a different number of neurons and relu as their activation function. The last dense layer had 6 neurons with softmax as its activation function to render the probability regarding 6 types of activity. Although many former studies used the concept of the CNN-LSTM hybrid model, the elements, including the number of layers, filters, LSTM cells, neurons, and dropout rate in the structure we proposed here, were determined by us. We implemented the 1D-CNN-LSTM model in the programming language Python 3.8.10 using a package called Tensorflow 2.5.0. We used the Adam optimizer for training our model with a learning rate of 0.001. We trained each model for 500 epochs using a batch size of 2000. After each epoch, we evaluated the performance of our model using the test data and saved the best model that showed the best accuracy on test data to calculate other evaluation metrics. The model can be reimplemented easily, and the results are completely reproducible. A detailed summary of the overall architecture is presented in Table 4.

## 3. Results

This section will discuss the validation procedure, evaluation metrics, data reshaping process, the effect of window length on the overall result using divergent evaluation metrics, and the effect of window length on each activity. 

### 3.1. Validation Procedure 

We accumulated data from 42 participants. We used the leave one out cross-validation method for our study, which we can also refer to as inter-participant evaluation. We had to train and test our model 42 times to execute this procedure. Each time, we had data from 41 participants in the training data and data from the other participant in the test data. Every time we trained and tested our model, we had data from a different participant in the test set. We were able to investigate the overall impact of our study on each participant.

### 3.2. Evaluation Metrics 

To evaluate the performance of our findings, we used four evaluation metrics: accuracy, precision, recall, and *F*1-measure. The definition and purpose of using each metric are given below:

#### 3.2.1. Accuracy 

Accuracy [41] is defined by the ratio of the correct number of predictions to the total number of predictions. It is well suited to the classification task where each class has an almost similar number of samples. It can be calculated using the following formula:

$$\mathrm{accuracy}=\frac{\mathrm{Number}\;\mathrm{of}\;\mathrm{correct}\;\mathrm{predictions}}{\mathrm{Total}\;\mathrm{number}\;\mathrm{of}\;\mathrm{predictions}}$$

#### 3.2.2. Precision 

This metric is used to identify if the model is equally capable of identifying each class. This metric is helpful to evaluate the model’s performance for each class separately. High precision for a class refers to the model’s efficient performance in identifying that class. Low precision for a class means that the model is not considered capable of recognizing that class. We can calculate precision [41] for any class A using the following formula:

$$\mathrm{Precision}=\frac{\mathrm{True}\;\mathrm{positives}}{\mathrm{True}\;\mathrm{positives}+\mathrm{False}\;\mathrm{positives}}$$

Here,

True positives = number of correctly predicted samples of class A;

False positives = number of samples predicted as class A but not belonging to class A.

#### 3.2.3. Recall

This metric is also suitable to evaluate models’ performance for each class separately. It helps determine if the model is pruned to misclassification for a particular class. High recall for a class means that the model is not pruned to misclassify that class as another class. Low recall for a class refers to the model’s proneness in misclassifying that class as another class. We use the following formula to calculate recall [41] for any class A:

$$\mathrm{Recall}=\frac{\mathrm{True}\;\mathrm{positives}}{\mathrm{True}\;\mathrm{positives}+\mathrm{False}\;\mathrm{negatives}}$$

Here,

True positives = number of correctly predicted samples of class A;

False negatives = number of samples not predicted as class A but belonging to class A.

#### 3.2.4. *F*1 Measure 

From the definition of precision and recall, precision emphasizes keeping the predictions accurate, whereas recall prioritizes increasing the number of correct predictions. For any model, we need to maintain a precision-recall trade-off, where we want to increase the number of correct predictions while keeping the predictions as accurate as possible. The *F*1 measure [42] represents a model’s ability to maintain proper precision and recall. It does so by computing a harmonic mean of precision and recall using the following formula:

$$F1-\mathrm{measure}=2\times \frac{\mathrm{Precision}\;\times \mathrm{Recall}}{\mathrm{Precision}+\mathrm{Recall}}$$

A model with a high *F*1 measure represents the model’s ability in maintaining both high recall and precision, whereas a low *F*1 measure represents the opposite.

### 3.3. Data Reshaping

CNN was the first part of our proposed architecture of 1D-CNN-LSTM. So, we initially reshaped the data to feed into CNN’s first convolution layer. As mentioned earlier, CNN works with a data matrix or data window. Each window may consist of a particular portion of data. This window helps CNN grasp knowledge about a current data point by providing some past data points or future data points. We can also refer to this data matrix as an image from where the CNN will extract features to learn more efficiently. Since the CNN requires a data window as its input, we had to construct windows of data from our whole dataset. The number of samples each window contains is called window length. The training and test sets need to be segmented into windows of equal length. Each consecutive window may have common training samples. The number of common training samples between each window depends on the overlapping ratio [43]. We can use the following formula to calculate the overlapping ratio:

$$\mathrm{Overlapping}\;\mathrm{Ratio}=\frac{\mathrm{Number}\;\mathrm{of}\;\mathrm{common}\;\mathrm{samples}\;\mathrm{between}\;\mathrm{two}\;\mathrm{consecutive}\;\mathrm{windows}}{\mathrm{Window}\;\mathrm{length}}$$

In our study, we wanted to observe how different window lengths influence the performance of the 1D-CNN-LSTM model in the case of HAR. To conduct this study, we recorded the performance of our model for different window lengths. We started by segmenting the training set, and test set into windows with a window length of 5 and then evaluated the model’s performance. We then raised the window length by 10 and re-recorded the model’s performance. We continued the process until we reached a window length of 195. We did not increase the window length further because it increased the training time considerably. The number of common samples between each consecutive window for a particular window length was window length−1. So, the overlapping ratio for a particular window length was:

$$\mathrm{Overlapping}\;\mathrm{Ratio}\;\mathrm{for}\;a\;\mathrm{particular}\;\mathrm{window}\;\mathrm{lentgh}=\frac{\mathrm{Window}\;\mathrm{length}-1}{\mathrm{Window}\;\mathrm{length}}$$

It should be mentioned that although each window had multiple samples, there was only one label (activity) that corresponded to each window. The label for each window was the activity corresponding to the last sample of that window. Details about the segmented datasets are illustrated in the Table 5 below.

### 3.4. Effects of Window Length on the Overall Result 

**Figure 8:**
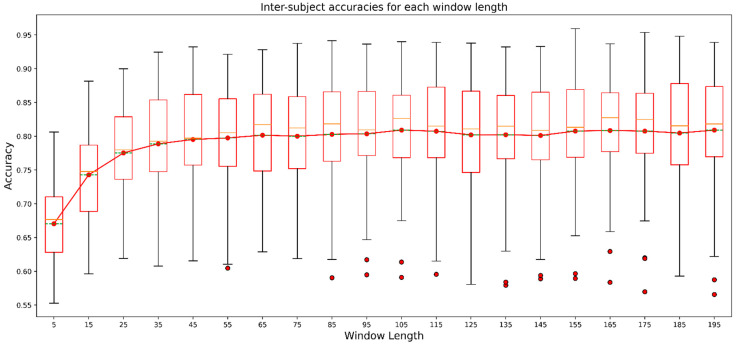
Box plot showing spreads of results for each participant for all window lengths. The red line connects the points indicating average accuracy at each window length. The red horizontal line in the bar denotes the median values whereas the green dashed horizontal line denotes the average values.

We averaged the accuracy for all the participants at each window length, and the average accuracy gradually increased until we reached a window length of 55. We can observe the effect of window length on average accuracy in Figure 8. At the lowest window length, which is 5, the average accuracy was 67.04%. At a window length of 55, the average accuracy was 79.74%, and as the window length became greater than 55, we could not see considerable change. For a window length from 65 to 195, the average accuracy was around 80%. We recorded the highest average accuracy for the window length of 105, which was 80.91%. It is clear that the window length influenced the model’s performance, but after a certain length, there was hardly any influence on the model’s performance. 

Furthermore, we can also observe the spread of accuracies for all the participants. The highest accuracy was about 81% for a particular participant at the lowest window length, and the lowest recorded accuracy was about 56%. However, as the window length increased, the highest recorded accuracy for any window length also increased. We recorded the highest accuracy, around 97% for a particular participant, for the window length of 155. Although the highest recorded accuracy for any window length increased with the increment of window length, the lowest recorded accuracy for any window length did not improve considerably. It seemed that the accuracy remained poor for some participants, even for bigger window lengths. This scenario can be explained better with participant-wise analysis.

### 3.5. Effect of Window Length on Model Performance for Individual Participants

**Figure 9:**
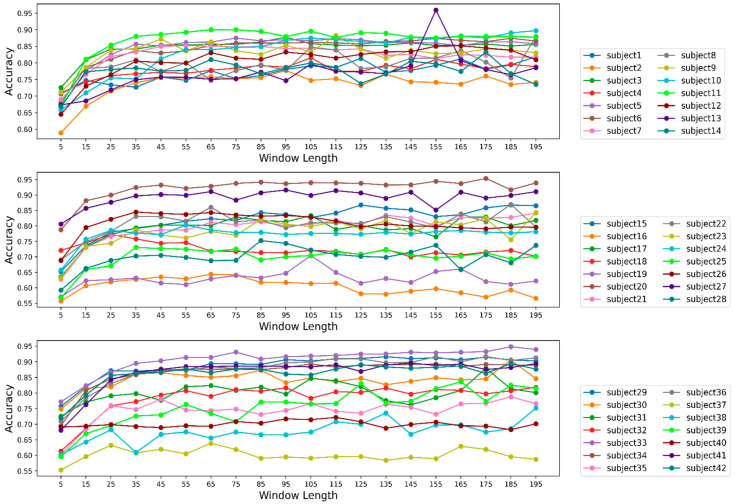
Accuracies for each participant for different window lengths.

From the previous section, we found out that the average accuracy for all the participants became steady with an increment of window length. We will now observe if the scenario was the same for the individual participant. We can determine the effect of window length for each participant from Figure 9. 

From Figure 9, we can see that the accuracy improved for the first 14 participants as the window length increased and remained steady after a window length of 55. Most of the participants showed a slight increment in the accuracy for a window length of 105. Among participants 1 to participant 14, the model performed best when we tested the model using the data from participant 11. We recorded the highest accuracy, around 97%, for participant 11 at a window length of 155; however, the performance was not consistent. For participants 15 to 28, the scenario was almost the same as we had for the first 14 participants, but for participant 16, the accuracy did not improve with window length; rather, we found a downward trend. We recorded the highest accuracy for participant 16 at a window length of 65, which was about 65%. Accuracy for participant 19 was found to be as poor as we saw for participant 16, although the accuracy for participant 19 had an increased accuracy of around 70% at a window length of 105. From participants 15 to 28, we recorded the best performance for participant 20 almost at every window length. Regarding the model’s performance for participants 29 to 42, we observed poor outcomes from the model for participant 37, which resembled the model performance for participant 16. The best performance from the model was recorded for participant 33 for almost every window length. 

### 3.6. Effect of Window Length on Model Performance for Each Activity

**Figure 10:**
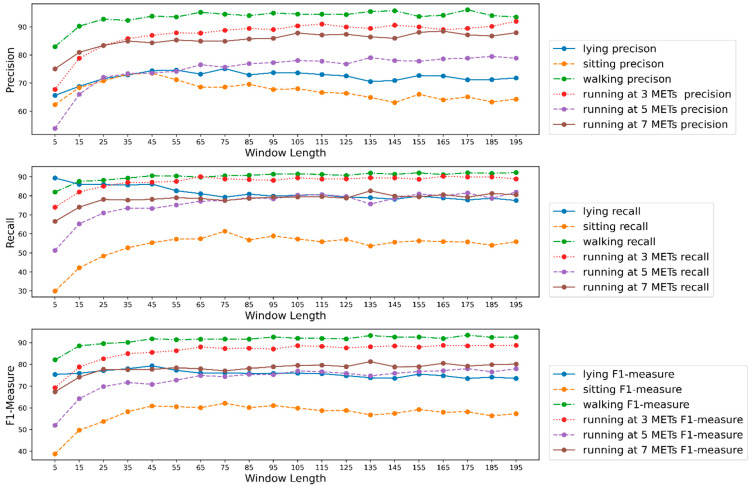
Precision, recall, and f1 measure for each activity class at different window lengths.

As mentioned earlier, we considered the metrics precision, recall, and *F*1 measure to evaluate the impacts of window length on each activity. We calculated precision for each activity class at each window length for all participants and averaged the precision as depicted in Figure 10. From Figure 10, we can see that precision increased until a certain window length for all six different activities. After a window length of 45, precision either remained steady or improved for all activities except for sitting. In addition, we experienced a high precision for high-intensity activities such as walking and running at 3 METs, 5 METs, and 7 METs. Whereas, the precision was comparatively poor for low-intensity activities such as lying and sitting. We recorded higher precision for activity walking than all other activities for every window length, which means that the models were highly accurate in predicting the activity walking. A more detailed scenario about precision is shown in Table 6. We can see the models’ highest average precision for each activity and the window length at which we recorded the highest precision in Table 6.

Regarding recall, we observed very poor recall for the activity sitting. Experiencing poor precision and recall for the activity sitting means that the models experienced difficulties correctly identifying it. It also means that the models misclassified many samples belonging to other activities such as sitting and many samples from the activity sitting as other activities. We recorded impressive recall for the activities walking and running at 3 METs. Recalls recorded for the other 3 activities were considerably decent. An interesting trend we observed for the activity lying is that the recall was about 90% at the lowest window length and the recall reduced as the window length increased. In contrast, recalls for all other activities increased until a certain window length and then became steady. A detailed numerical description of recall is given in Table 6.

The *F*1 measure depicted similar trends as precision and recall. We recorded a high *F*1 measure for walking and running at 3 METs. We had a decent *F*1 measure for all other activities except for sitting. The *F*1 measure was very poor for the activity sitting. *F*1 measures increased until a certain window length for all the activities and remained steady as window length increased. We also provide a numerical description of the *F*1 measure in Table 6.

## 4. Discussion

**Figure 11:**
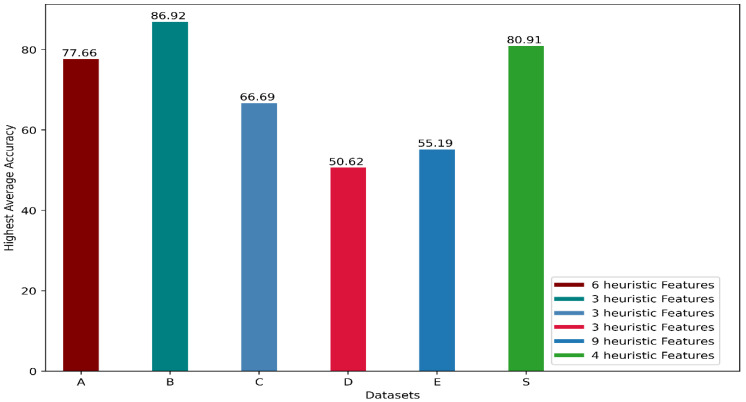
The highest accuracies achieved for datasets A, B, C, D, and E with the different numbers of heuristic features in [10], along with the accuracy we found for our dataset denoted by S using the 4 most significant heuristic features.

In [10], researchers studied the performance of heuristic features for five publicly available datasets, which they labeled as A [44], B [45], C [21], D [46], and E [47]. Besides using the 9 features altogether, they also used only the first 3 or the first 6 heuristic features and recorded the performance of 4 classifiers, Bayesian decision-making (BDM), K-nearest neighbor (KNN), support vector machine (SVM), and artificial neural network (ANN). They used 10-fold cross-validation technique where each fold contained data for a particular participant. We can call our used inter-participant validation technique a 42-fold cross-validation technique where each fold contains data for a particular participant. They recorded accuracy from each classifier using the first 3 heuristic features, the first 6 heuristic features, and all 9 heuristic features. They achieved the highest accuracy for datasets B, C, and D using the first three heuristic features. To acquire the highest accuracy for datasets A and E, they used the first 6 and all 9 heuristic features, respectively. For all the datasets, they found the best performance using SVM. From their results, it was clear that all 9 heuristic features were not critically important to model performance since, for 3 of their datasets, they recorded the best performance using only the first 3 heuristic features. However, they did not try all the combinations of features, and there was no analysis to select the most significant features. We performed that analysis and found the first four features to be the most important. We plotted the highest accuracy they achieved using the heuristic features for each dataset and also indicated the number of features for which they found the best performance in Figure 11. We also included the highest average accuracy we achieved, using the four most important features we found in the plot to provide a comparative perspective.

Although it is not feasible to compare our result with the results found in [10] since they used different classifiers and datasets, we acquired results comparable to their performance, even with more participants than they had for an inter-participant validation method. However, our main objective was to explore the effects of window length in HAR execution for 1D-CNN-LSTM.

Many studies have explored HAR using deep neural networks like CNN, LSTM, or hybrids, but few studies in HAR have reported the effects of window length or time steps. Most of the studies chose the time steps or window length, claiming that they achieved the best performance using that particular window length. For instance, [48] used a CNN and gated recurrent unit (GRU) hybrid on three datasets named UCI-HAR, WISDM, PAMAP2, and acquired 96.20%, 97.21%, 95.27%, respectively. Still, they did not mention how they chose the window length of 128 for their model. In another study [49], which used the same dataset as in [48], they also did not mention the reason behind choosing 128 as their window lengths; rather, they emphasized their proposed CNN, bidirectional LSTM hybrid-model architecture and acquired accuracies. Many other studies [13,50,51,52] explored divergent forms of deep learning architectures using the popular UCI-HAR dataset and used the same window length of 128 samples. Another study [53], using a dataset called “Complex human activities using smartphones and smartwatch sensors”, explored the performance of divergent deep neural networks including LSTM, bidirectional LSTM, GRU, bidirectional GRU, CNN-LSTM, CNN-BiLSTM, CNN-GRU, and CNN-BiGRU for five different window lengths (in seconds) of 5, 10, 20, 30, and 40 s. They achieved the highest accuracy of 98.78% using CNN-BiGRU when they used the window length of 40 s. However, exploring only five different window lengths was insufficient to depict the influence of window lengths. Therefore in our study, we explored the performance of 1D-CNN-LSTM for 19 different window lengths. We can observe from Figure 8 that the recorded results showed that window length had a significant impact on the performance of the models in HAR. However, the impact was noticeable until we reached a window length between 55 to 85 in the study. After that, the performance was not influenced substantially by incremental increases in window length. We can call the window length range of 55 to 85 the saturation range for the models’ performance. The reason behind such a trend could be that, after a certain length, even if we increase the window length, the model could not extract significant knowledge to enhance its performance. Although Figure 8 displays the averaged effect of window length, we observe the influence on individual participants in Figure 9. We experienced a similar trend for almost all participants. We had a contradictory trend in performance for participants 16 and 37. After the saturation range with the increment in window length models, performance for those participants was reduced. Although the decrement was not considered important, it was not usual if we observe the performance trend for other participants. This could happen due to noisy samples in the datasets belonging to those two participants, which need to be further analyzed. Observing the effect of window length on each activity class, we found that precision, recall, and f1-measure had very poor values for the sitting activity. In addition, the values of evaluation metrics reduced with an increase in window length for lower-intensity activities like sitting and lying. For instance, recall for lying was about 90% when the window length was the smallest but as the window length increased, the recall decreased. The effect of window length on lower-intensity activities was an interesting observation which was not evaluated in previous studies. We can assume that window length had different effects for activities with different intensities considering our outcome. When choosing a window length, we should also consider a window length that will help generate a better outcome for lower- and higher-intensity activities. Another reason behind such poor performance could be the lower number of samples for the activity sitting which we can see in Table 3. Balancing the classes could have helped to improve the situation. Still, we did not do it in our study as our main objective was to observe the influence of window length rather than increasing the models’ performance. 

From Table 6, we can see that for most of the activity classes, the highest metric values we found were when the window length was above 150, but the highest values were not considerably greater than the values we found at saturation point. That means we need not choose a very high window length to achieve the best performance from the model; rather, we need to select a window length around the saturation range that will be considerably smaller than the window lengths where we found the highest metric values. If we manage to keep the window length smaller, it would reduce the time complexity for the models and increase the computational efficiency. 

Although we have conducted analysis participant-wise and activity-wise, some analysis is yet to be done. For instance, we achieved very high performance for some participants, such as participants 26, 27, and 33 and very poor performance for participants 16, 19, and 37. Still, we did not try to determine why the model performed differently, especially for these participants; as we mentioned earlier, our objective was to study the effect of window length in 1D-CNN-LSTM in HAR. 

In brief, we found that window length in 1D-CNN-LSTM had a significant effect on HAR. We found that the training time was affected by the window length. As the window length increased, the training time also increased. The approximate training time for the model using the lowest window length of 5 was about 40 minutes and almost 20 hours for the highest window length of 195. For our suggested saturation range of 55 to 85, the training time was about 4 hours. Here, we approximated the mentioned training time for each iteration of inter-subject validation, which means there were data from 41 subjects in training data, and test data included data from one subject, which we did not include in the training data. So, window length should not be arbitrarily long; rather, it should be chosen wisely by correctly identifying the saturation range so that the model offers less time complexity while training and more efficiency. In addition, for the 1D-CNN-LSTM model, other studies may choose a window length from our suggested saturation range of 55 to 85 for HAR. We resampled the whole dataset to 30 Hz, so our proposed saturation range should be 1.83 s to 2.83 s. 

There are a number of limitations to our current study. We only used one accelerometer for our study to keep the computation complexity low as we conducted inter-participant validation for 42 participants. However, we may experience improvements in our study if a gyroscope sensor was also used with the accelerometer sensor since a gyroscope sensor can provide substantial information regarding the rotational nature. Moreover, the data were collected from only one position, a phone in the pocket, and we did not study how much the analysis would be affected if we used data from different body parts. In addition, we studied the effect of window length on one type of model, but other models also take windows of data as an input. We do not know if the effect would be the same for those models. However, we initiated this type of analysis using many participants, one accelerometer sensor, data from one position, and only one type of model. In future, we will try to conduct the same study using different models and settings.

## 5. Conclusions

Our study wanted to depict the influence of window length in 1D-CNN-LSTM on HAR. We used a large dataset accumulated from 42 participants for 6 different activities. The samples were from an accelerometer sensor in a smartphone kept in the pocket. We used four heuristic features to eliminate variations produced due to the rotation of the smartphone. We found a saturation range for window length, after which the model’s performance was not considerably influenced by the window length. 

## Figures and Tables

**Table 1:** Demographics of the participants.

Number of Participants		Age (Years)			Height (cm)			Weight (kg)		
Male	Female	Average	Maximum	Minimum	Average	Maximum	Minimum	Average	Maximum	Minimum
18	24	29	56	18	169.17	185	143	68.19	95.2	43

**Table 2:** Duration and order of activities performed by each participant.

Rank	Activity	Duration (Minutes)
1	Lying down	5
2	Sitting	5
3	Walking	10
4	Lying down	5
5	Running at 3-METs	10
6	Lying down	5
7	Running at 5-METs	10
8	Sitting	5
9	Running at 7-METs	10

**Table 3:** Number and ratio of samples for each type of activity class.

Activity	Number of Data Points	Ratio to Total Dataset
Running at 7-METs	926,606	21.43%
Running at 5-METs	812,135	18.78%
Running at 3-METs	815,498	18.86%
Walking	609,406	14.09%
lying	696,329	16.10%
sitting	464,559	10.74%

**Table 4:** A detailed description of our proposed 1D-CNN-LSTM model.

Parts of Architecture	Components of Each Part (Blank Cell = Not Available for This Layer)							
CNN	**Layer’s Name**	**Number of Filters**	**Kernel Size**	**Activation Function**	**Dropout Ratio**	**Pooling Type**	**Pool Size**	**Padding Type**
	Convolution	512	5	relu				same
	Dropout				0.3			
	Pooling					Average	3	same
	Convolution	256	3	relu				same
	Dropout				0.3			
	Convolution	64	3	relu				same
	Pooling					Average	3	same
	Convolution	128	3	relu				same
	Convolution	256	5	relu				same
	Dropout			N/A	0.3			
	Convolution	512	7	relu				same
	Dropout				0.3			
	Pooling					Average	3	same
LSTM	**Layer’s Name**			**Number of Units**		**Activation Function**		
	LSTM			512		tanh		
Fully Connected Network	**Layer’s Name**			**Number of Neurons**		**Activation Function**		
	Dense			100		relu		
	Dense			28		relu		
	Dense			64		relu		
	Dense			6		softmax		

**Table 5:** The number of windows in the training set and the test set for each window length.

Window Length	Overlapping Ratio (%)	No. of Windows in the Training Set ± Standard Deviation	No. of Windows in the Test Set ± Standard Deviation
5	80.00	4,221,561 ± 31,399	102,959 ± 31,399
15	93.33	4,221,551 ± 31,399	102,949 ± 31,399
25	96.00	4,221,541 ± 31,399	102,939 ± 31,399
35	97.14	4,221,531 ± 31,399	102,929 ± 31,399
45	97.77	4,221,521 ± 31,399	102,919 ± 31,399
55	98.18	4,221,511 ± 31,399	102,909 ± 31,399
65	98.46	4,221,501 ± 31,399	102,899 ± 31,399
75	98.66	4,221,491 ± 31,399	102,889 ± 31,399
85	98.82	4,221,481 ± 31,399	102,879 ± 31,399
95	98.94	4,221,471 ± 31,399	102,869 ± 31,399
105	99.04	4,221,461 ± 31,399	102,859 ± 31,399
115	99.13	4,221,451 ± 31,399	102,849 ± 31,399
125	99.20	4,221,441 ± 31,399	102,839 ± 31,399
135	99.25	4,221,431 ± 31,399	102,829 ± 31,399
145	99.31	4,221,421 ± 31,399	102,819 ± 31,399
155	99.35	4,221,411 ± 31,399	102,809 ± 31,399
165	99.39	4,221,401 ± 31,399	102,799 ± 31,399
175	99.42	4,221,391 ± 31,399	102,789 ± 31,399
185	99.46	4,221,381 ± 31,399	102,779 ± 31,399
195	99.49	4,221,371 ± 31,399	102,769 ± 31,399

**Table 6:** Highest and lowest averaged precision, recall, and f1 measure for each activity and respective window length.

Activities	Properties for the Highest Precision		Properties for the Lowest Precision		Properties for the Highest Recall		Properties for the Lowest Recall		Properties for the Highest *F*1 Measure		Properties for the Lowest *F*1 Measure	
	Highest Value	Window Length	Lowest Value	Window Length	Highest Value	Window Length	Lowest Value	Window Length	Highest Value	Window Length	Lowest Value	Window Length
Lying	76.16	75	65.63	5	89.38	5	77.58	195	79.30	45	73.55	175
Sitting	73.53	45	62.34	5	61.41	175	29.91	5	62.15	75	38.74	5
Walking	96.10	175	82.99	5	92.29	195	82.00	5	93.46	175	82.07	5
Running 3 METS	91.98	195	67.73	5	90.26	165	74.04	5	88.76	195	69.30	5
Running 5 METS	79.48	185	53.87	5	81.99	195	51.30	5	78.08	175	51.95	5
Running 7 METS	88.49	165	75.08	5	82.62	135	66.57	5	81.26	135	67.39	5

## Data Availability

The data presented in this study are available on request from the corresponding author. The data are not publicly available due to ethical constraints.
